# Integrative analysis of hub genes and key pathway in two subtypes of diffuse large B‐cell lymphoma by bioinformatics and basic experiments

**DOI:** 10.1002/jcla.23978

**Published:** 2021-09-21

**Authors:** Qian Li, Ye Meng, Linhui Hu, Alice Charwudzi, Weiwei Zhu, Zhimin Zhai

**Affiliations:** ^1^ Department of Hematology/Hematological Lab The Second Hospital of Anhui Medical University Hefei China

**Keywords:** diffuse large B‐cell lymphoma, expression profiling data, hub genes, prognosis, signaling pathway

## Abstract

**Background:**

The germinal center B‐cell (GCB) and activated B‐cell (ABC) subtypes of diffuse large B‐cell lymphoma (DLBCL) have a significant difference in prognosis. This study aimed to identify potential hub genes, and key pathways involved in them.

**Methods:**

Databases including Gene Expression Omnibus (GEO), Gene Ontology (GO), Kyoto Encyclopedia of Genes and Genomes (KEGG), and STRING were accessed to obtain potential crucial genes and key pathways associated with the GCB and ABC. Then qRT‐PCR and Western blot experiments were performed to verify the most clinically significant gene and pathway.

**Results:**

Three cohort datasets from the GEO database were analyzed, including 195 GCB and 169 ABC samples. We identified 1113 differentially expressed genes (DEGs) between the GCB and ABC subtypes. The DEGs were mainly enriched in biological processes (BP). The KEGG analysis showed enrichment in cell cycle and Wnt signaling pathways. We selected the top 10 genes using the STRING database and Cytoscape software. We used 5 calculation methods of the cytoHubba plugin, and found 3 central genes (IL‐10, CD44, CCND2). CCND2 was significantly related to the prognosis of DLBCL patients. Besides, our experimental results demonstrated a significantly higher expression of CCND2 in the ABC‐type cell line than in the GCB‐type; it was proportional to the expression of key proteins in the Wnt signaling pathway.

**Conclusion:**

CCND2 overexpression and Wnt pathway activation might be the main reasons for the poor prognosis of ABC‐DLBCL.

## Graphical abstract

The study revealed that CCND2 overexpression and Wnt pathway activation may be factors of the poor prognosis of ABC‐subtype DLBCL by integrative bioinformatic analysis and basic experiments.

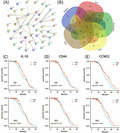

## 1 INTRODUCTION

Diffuse large B‐cell lymphoma (DLBCL) accounts for about 30% of all lymphoma cases; it is the most common malignant tumor of the lymphatic system.^1^ According to the cell of origin classification, DLBCL has two major biologically distinct molecular subtypes, namely the GCB (germinal center B‐cell) and ABC (activated B‐cell).^2^ ABC‐DLBCL has worse clinical outcomes when treated with standard chemoimmunotherapy.^3^, ^4^ However, the biological mechanisms are still controversial. The molecular classification not only predicts prognosis but also relates to the personalized therapy of DLBCL patients, so novel therapeutic targets and effective treatment options need to be ascertained.

In previous studies, some focused on genomic damages and treatment resistance. Some indicated that ABC‐DLBCL seems to freeze in the late state, showing apparent plasma cell differentiation arrest.^5^, ^6^ The results may have limitations due to tissue or sample heterogeneity. Since the prognosis of patients with two subtypes of one disease is unexpectedly different, is there any relationship with cell cycle or tumor stemness between them? With the rapid development of gene sequencing technologies, we could explore it via combining bioinformatics methods with expression profiling even basic experiments.^7^ Regardless, it undoubtedly provides convenience for our scientific research. Here, we selected three microarray datasets to facilitate our analyses. After selecting intersecting genes to improve accuracy, we performed gene function and pathway enrichment analysis. A series of analytical tools and software were applied to obtain information such as hub genes, signal pathways, and survival periods. Most related studies focused on one cohort, but sequencing results are often limited and inconsistent due to samples' heterogeneity in independent studies.^6^, ^8^ Therefore, it is necessary to apply integrated bioinformatics methods on quality and merged expression profiles to increase statistical power in detecting more reliable genes. More importantly, the above results need to be verified further by experiments in vitro. However, no such study has been reported so far. Our analysis included this comparison, and it made us understand the mechanism better and reveal the difference in molecular expression between the two subtypes.

Due to the different pathogenesis and prognosis of survival, it is urgently vital to clarify the etiology and molecular mechanisms of these DLBCL subtypes and to determine molecular biomarkers for diagnosis and individualized treatment.

## 2 MATERIALS AND METHODS

### 2.1 Cell culture

In this study, we used two human diffuse large B‐cell lymphoma cell lines, including one GCB subtype (SU‐DHL‐6) and one ABC subtype (SU‐DHL‐2) cell line. They were purchased from Saiku Company. Both were cultured in RPMI‐1640 complete medium (Gibco), supplemented with 10% fetal bovine serum (Gibco), and incubated at 37℃ in a 5% CO_2_ incubator.

### 2.2 Data source

The datasets we analyzed came from the GEO database (Gene Expression Omnibus) (http://www.ncbi.nlm.nih.gov/geo/), which were chosen after searching for keywords related to GCB and ABC subtype of DLBCL on the same platform (GPL570, [HG‐U133 Plus 2] Affymetrix Human Genome U133 Plus 2.0 Array). We retrieved 232 DLBCL molecular subtype series based on the cell of origin algorithm from the GEO database. Three independent datasets (GSE19246,^9^ GSE87371,^10^ and GSE56313^11^) were selected for the research. From these profiles, we retrieved information about 364 individual DLBCL samples.

### 2.3 Data processing of DEGs

The GEO2R online analysis tool of the National Institute of Biomedical Research (https://www.ncbi.nlm.nih.gov/geo/geo2r/) was used to find the differentially expressed genes (DEGs) between the ABC and GCB subtypes. The genes that met the cut‐off criteria (*p* < 0.05 and |logFC(fold change)| ≥ 1.0 after adjustment) were considered DEGs. Statistical analysis was performed on all datasets. The online VENN diagram tool was used to identify intersecting genes (Bioinformatics.psb.ugent.be/webtools/venn/).

### 2.4 Functional enrichment analyses of DEGs

The GO (Gene Ontology) annotation and the KEGG (Kyoto Encyclopedia of Genes and Genomes) pathway enrichment analyzes of DEGs were performed with the DAVID database (Database for Annotation, Visualization and Integrated Discovery, https://david.ncifcrf.gov/)^12^. GO is used to understand the biological functions, pathways, or positioning of DEGs. The three main GO terms analyzed were the biological processes (BP), cellular components (CC), and molecular functions (MF). KEGG is used to collect information on molecular biological pathways. The cut‐off criteria were *p* < 0.1 and gene count ≥2. Furthermore, ggplot2 and clusterProfiler packages of R software (version 3.6.3; http://bioconductor) were used to perform the GO, and KEGG analyzes and visualization.^13^

### 2.5 PPI network integration and Hub gene identification

The DEGs data were uploaded to the Search Tool for the Retrieval of Interacting genes (STRING) database^14^ to construct a network based on protein‐protein interaction (PPI). Confidence score >0.4 was set, and the resulting output was used in the Cytoscape software (www.cytoscape.org/) to visualize the network and select hub proteins.^15^ We utilized five calculation methods (Degree, EPC, Eccentricity, MCC, and MNC) of the CytoHubba application to select the top 10 genes. The central (intersecting) genes were considered core candidate genes, as nodes with a higher degree of connection are more significant and likely to influence biological function. Then, the top 10 genes from each method were uploaded to the online website (http://www.Ehbio.com/ImageGP/index.php/Home/Index/VennDiagram.html) to obtain the intersecting genes, representing key candidate genes with essential biological regulatory functions.

### 2.6 Survival analysis

We performed survival analysis on the intersecting genes using the Survival and survminer R software packages (version 3.6.3; http://bioconductor). The normalized GSE87371 expression dataset was used for the survival analysis; the intersecting genes were divided into two subgroups based on their expression levels. Statistically significant OS and PFS were visualized with the R. Log‐rank *p* < 0.05 was considered significant.

### 2.7 Quantitative real‐time polymerase chain reaction

We isolated total RNA from each cell line using TRIzol reagent (Invitrogen), then Transcript RT Kit (Sangon) was used to transcribe the RNA into cDNA. The quantitative real‐time PCR (qRT‐PCR) was performed on an ABI 7500 Real‐Time PCR System (Life) with an SYBR Green Master Mix (TaKaRa). All the mRNA expressions were quantified based on the 2^ΔΔ^ *^C^* ^t^ method; GAPDH expression was used as an endogenous reference. The primers used are presented in Table 1.

**TABLE 1:** Sequences used for qRT‐PCR primers

Name	Sequences (5′ to 3′)
GAPDH—Forward	GTGAAGGTCGGTGTGAACGG
GAPDH—Reverse	GA TGCAGGGA TGA TGTTCTG
CCND2—Forward	ATCCGCAAGCATGCTCAGA
CCND2—Reverse	GATCATCGACGGTGGGTACAT

### 2.8 Western blot analysis

Cells (2 × 10^6^) were lysed in RIPA lysis buffer (Beyotime Biotechnology) and quantitated using a BCA Protein Assay Kit (Beyotime Biotechnology). The protein liquid was mixed with 5 × loading buffer in a volume ratio of 4 to 1, and placed in a boiling water bath for 10 min to denature. For Western blot analysis, equivalent amounts of protein per sample were electrophoretically resolved on 10% polyacrylamide gels and then transferred onto PVDF membranes (Millipore). Following this, the PVDF membranes with the protein were blocked with blocking buffer (Beyotime Biotechnology), then incubated with the corresponding primary antibodies. After repeated washes, the membranes were incubated with horseradish‐peroxidase‐conjugated anti‐mouse or anti‐rabbit secondary antibody (Cell Signaling, 1:1000 diluted) at room temperature for 1 h. An electro‐chemiluminescence (ECL) system (Thermo Fisher Scientific) was used for the detection. Anti‐β‐actin (Epitomics) was used to check for equal loading of protein between wells. The Primary antibodies used for the Western blot are shown in Table 2.

**TABLE 2:** Primary antibodies used for Western blot

Name	Company	Item number	Dilution ratio
CCND2	Cell signaling technology	#2978	1:1000
β‐catenin	Cell signaling technology	#2978	1:1000
WNT‐3α	Cell signaling technology	#2721	1:1000
Mouse anti‐βActin mAb	ZSGB‐BIO	TA‐09	1:1000

### 2.9 Statistical analysis

Statistical analyzes and graphing were performed with SPSS v19.0 (SPSS Inc.) and R statistical software, respectively. We utilized the log‐rank test to compare OS and PFS among patients in different groups. Student's t test were used to compare two groups of cell‐level experiments. Data were reported as mean ± SD (standard deviation). And *p*‐value <0.05 was regarded as statistically significant.

## 3 RESULTS

### 3.1 Identification of DEGs

**FIGURE 1:**
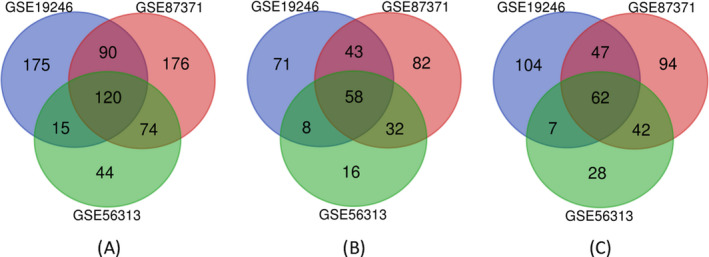
Venn diagram of differentially expressed genes (DEGs) shared in three Gene Expression Omnibus (GEO) datasets: GSE19246 (blue), GSE87371 (red), GSE56313 (green). (A) 120 DEGs were identified in three datasets. (B) 58 upregulated genes. (C) 62 downregulated genes. DEG identification criteria were adj. *p* < 0.05 and |logFC (fold change)| ≥ 1

As shown in Table 3, the GSE19246 dataset included 81 GCB subtype samples and 63 ABC subtype samples; GSE87371 contained 85 GCB subtype samples and 83 ABC subtypes samples; GSE56313 included 29 GCB subtype samples and 23 ABC subtype samples. Therefore, we integrated these datasets in subsequent processing steps. Using *p* < 0.05 and |logFC| ≥ 1 as the standard, a total of 1113 DEGs were identified between GCB and ABC subtypes of DLBCL, including 509 upregulated genes and 604 downregulated genes. In the GSE19246 expression profile, 400 DEGs were identified, of which 180 genes were upregulated, and 220 genes were downregulated. From the GSE87371 dataset, 460 DEGs were found, including 215 upregulated genes and 245 downregulated genes. From the GSE56313 dataset, 253 DEGs were found, of which 114 were upregulated genes and 139 were downregulated genes. Subsequently, we performed a VENN analysis and got the intersecting DEGs. In total, 120 DEGs were obtained after the intersection of all three groups (Figure 1A), of which 58 were upregulated genes (Figure 1B) and 62 were downregulated genes (Figure 1C).

**TABLE 3:** Statistics of three microarray databases originated from GEO database

Dataset ID	GCB	ABC	Total number
GSE19246	81	63	144
GSE87371	85	83	168
GSE56313	29	23	52

### 3.2 Enrichment analyses of DEGs

**FIGURE 2:**
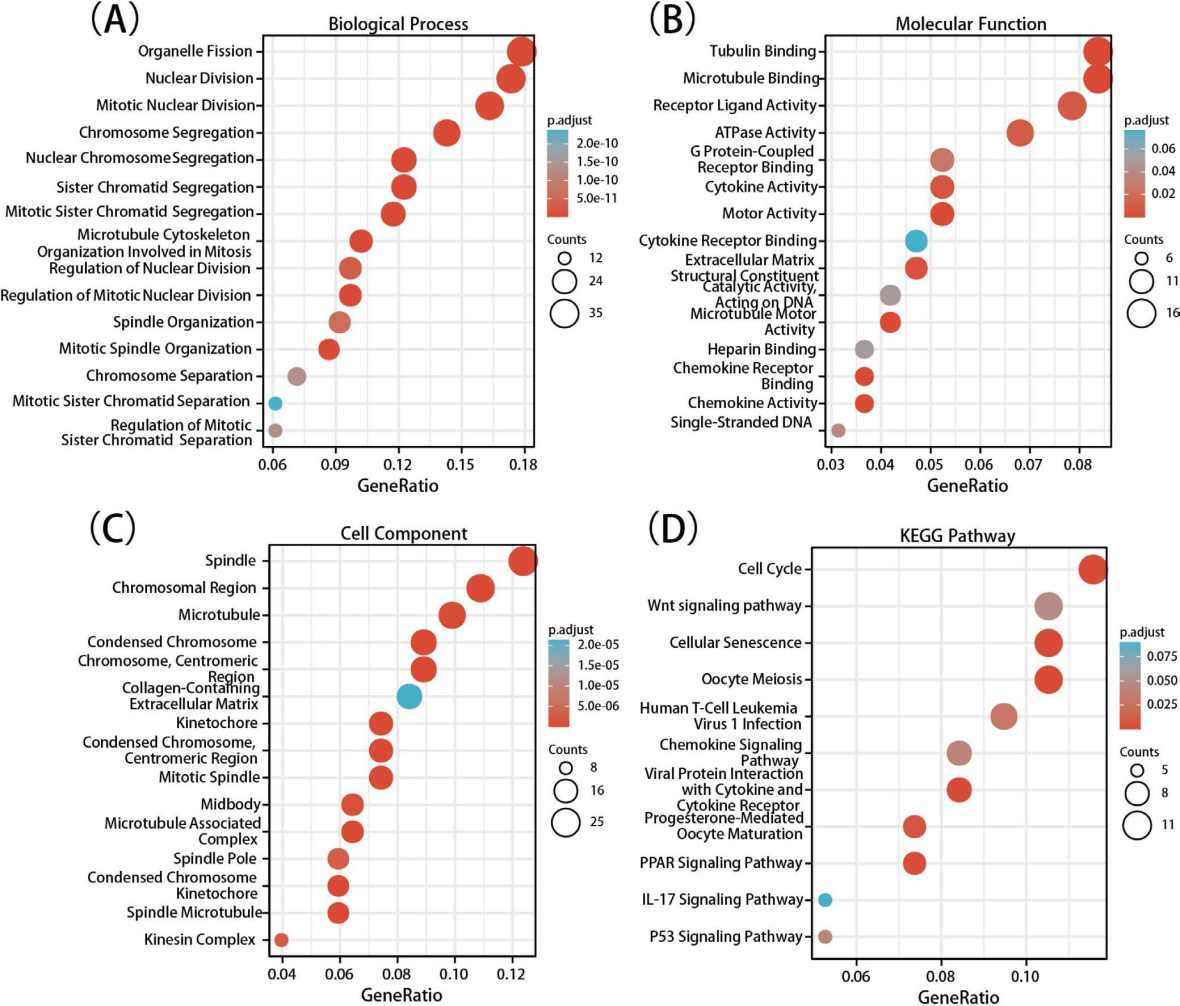
Significant enrichments of Gene Ontology (GO) terms and Kyoto Encyclopedia of Genes and Genomes (KEGG) pathways for differentially expressed genes (DEGs). (A) Enrichment of biological processes. (B) Enrichment of molecular functions. (C) Enrichment of cellular components. (D) Enrichment of KEGG pathway. Cut‐off criteria were *p* < 0.1 and gene count ≥ 2

We evaluated the functions and pathway enrichment of the candidate DEGs at the DAVID website. As shown in Figure 2, in GO analysis, the DEGs were mainly enriched in BP terms, including organelle fission, mitotic nuclear division, and so on (Figure 2A). In the MF group, receptor ligand activity and protein binding were the major enrichments (Figure 2B). Chromosome and mitotic spindle were mainly enriched in the CC terms (Figure 2C). The KEGG analysis showed enrichments mostly in cell cycle and Wnt signaling pathways (Figure 2D).

### 3.3 PPI network integration and Hub gene identification

**FIGURE 3:**
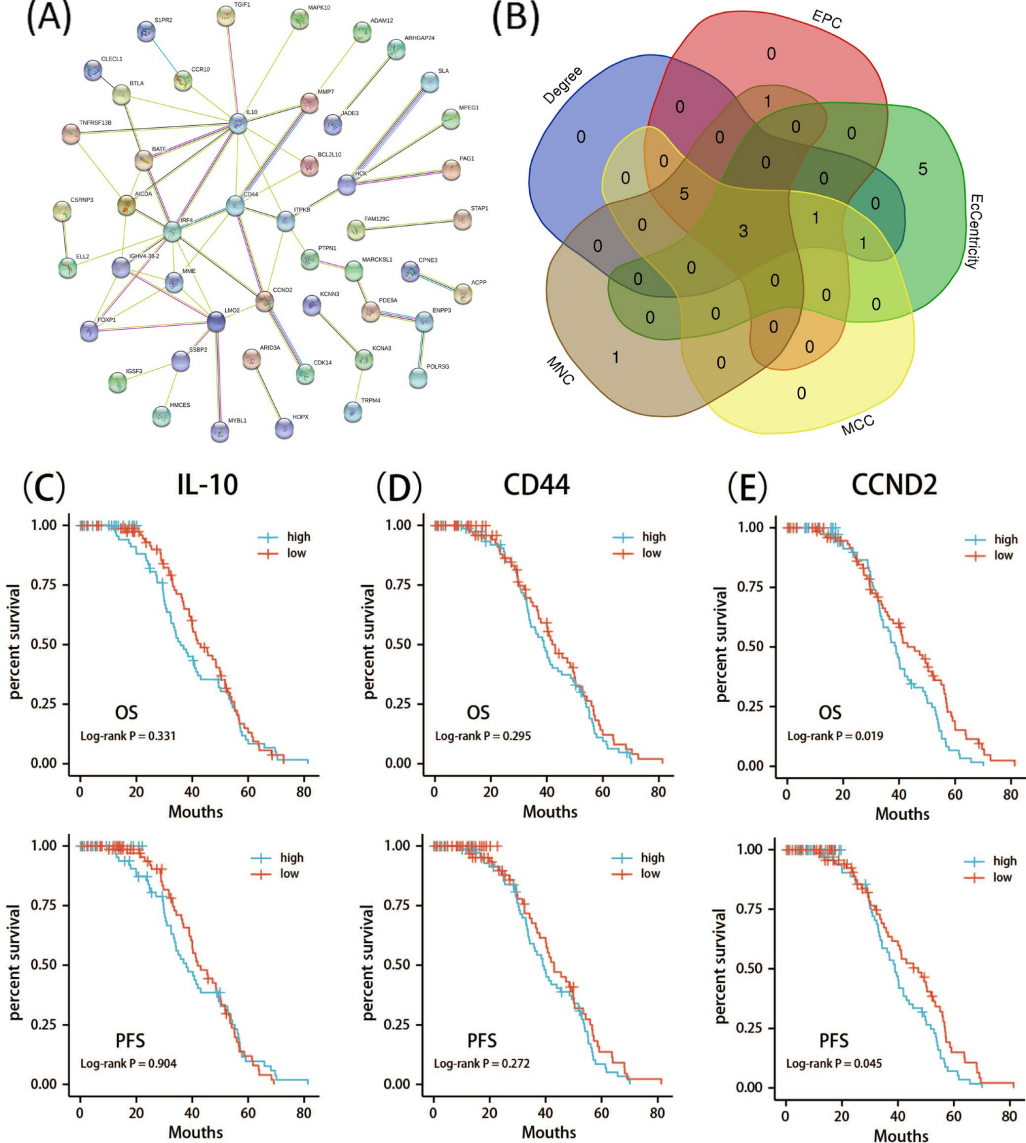
(A) Protein‐protein interaction network constructed with differentially expressed genes (DEGs), confidence score >0.4. (B) Venn plot of significant hub genes using five intersecting algorithms: Degree (blue), EPC (red), Eccentricity (green), MCC (yellow), MNC (brown). Areas with different colors correlate to different algorithm, the cross area included 3 hub genes (CCND2, CD44 and IL‐10), indicating the commonly accumulated DEGs. (C‐E) Association between 3 hub genes with over survival (above) and progress‐free survival (below) (C, IL‐10; D, CD44; E, CCND2) in those with DLBCL. High‐and low‐risk groups are shown in blue and red, respectively. *p* < 0.05 was the significance threshold

To better understand the interactions among the intersecting DEGs obtained from the three datasets, the STRING database was used to generate a PPI network consisting of 114 nodes and 62 edges, with parameters set to interaction score >0.4 and query proteins only being revealed (Figure 3A). The Cytoscape software was used to analyze hub proteins after importing the data, and the top 10 genes were evaluated with five calculation methods in the CytoHubba application (Table 4). Furthermore, we uploaded the genes from the five calculation methods to the Venn Diagram online website to generate intersecting genes to identify significant hub genes (Figure 3B). We obtained 3 intersected hub genes (CCND2, CD44, and IL‐10), indicating the common DEGs. As shown in Table 5, the above 3 hub gene expressions in each dataset were upregulated in the ABC subtype of DLBCL.

**TABLE 4:** Top 10 genes utilized in the PPI network using five calculation methods in Cytoscape

Gene name	Degree score	Gene name	EPC score	Gene name	Eccentricity score	Gene name	MCC score	Gene name	MNC score
IL‐10	12	IRF4	9.921	PTPN1	0.1542	IL‐10	12	IRF4	9
IRF4	10	IL‐10	9.627	ITPKB	0.1542	IRF4	10	IL‐10	9
CD44	7	CD44	9.002	CD44	0.1285	CD44	7	CD44	7
LMO2	7	AICDA	8.132	HCK	0.1285	LMO2	7	LMO2	5
CCND2	5	LMO2	8.057	CCND2	0.1285	CCND2	5	IGHV4‐38‐2	5
IGHV4‐38‐2	5	MME	8.005	IL‐10	0.1285	IGHV4‐38‐2	5	AICDA	5
AICDA	5	IGHV4‐38‐2	7.808	MARCKSL1	0.1285	AICDA	5	MME	5
MME	5	CCND2	7.57	BCL2L10	0.1101	MME	5	CCND2	4
ITPKB	5	BATF	7.233	PAG1	0.1101	ITPKB	5	BATF	4
HCK	4	ITPKB	7.205	MMP7	0.1101	HCK	4	FOXP1	4

**TABLE 5:** The expression characteristics of three hub genes in the intersecting region of the petal map

Gene	adj. *p*. Val	*p* Value	logFC	Dataset ID
CCND2	1.63E‐15	2.63E‐18	2.41	GSE87371
	3.20E‐06	9.26E‐09	1.59	GSE19246
	4.65E‐05	4.93E‐08	1.86	GSE56313
CD44	1.92E‐05	7.01E‐07	1.20	GSE87371
	2.16E‐05	9.24E‐08	1.60	GSE19246
	1.55E‐03	7.47E‐06	1.68	GSE56313
IL‐10	5.61E‐04	3.97E‐05	1.03	GSE87371
	1.03E‐03	9.82E‐06	1.28	GSE19246
	1.85E‐02	3.05E‐04	1.24	GSE56313

### 3.4 CCND2 may be a key prognostic gene in DLBCL

To investigate the impact of the 3 hub genes on the prognosis of DLBCL patients, we obtained data on the expression levels of the genes from the GSE87371 dataset and analyzed the survival time of patients with GCB and ABC subtypes. Using Survival and survminer packages of R software, we found higher expression of these 3 hub genes correlated with worse OS and PFS. This finding suggests the prognosis is worse for ABC subtype patients than GCB subtype patients. The survival analysis outcomes were IL‐10 (OS, log‐rank *p* = 0.331; PFS, log‐rank *p* = 0.904), CD44 (OS, log‐rank *p* = 0.295; PFS, log‐rank *p* = 0.272), CCND2 (OS, log‐rank *p* = 0.019; PFS, log‐rank *p* = 0.045) (Figure 3C,D,E). Notably, the expression level of CCND2 significantly correlated with the prognosis, both OS and PFS.

### 3.5 Experimental validation

**FIGURE 4:**
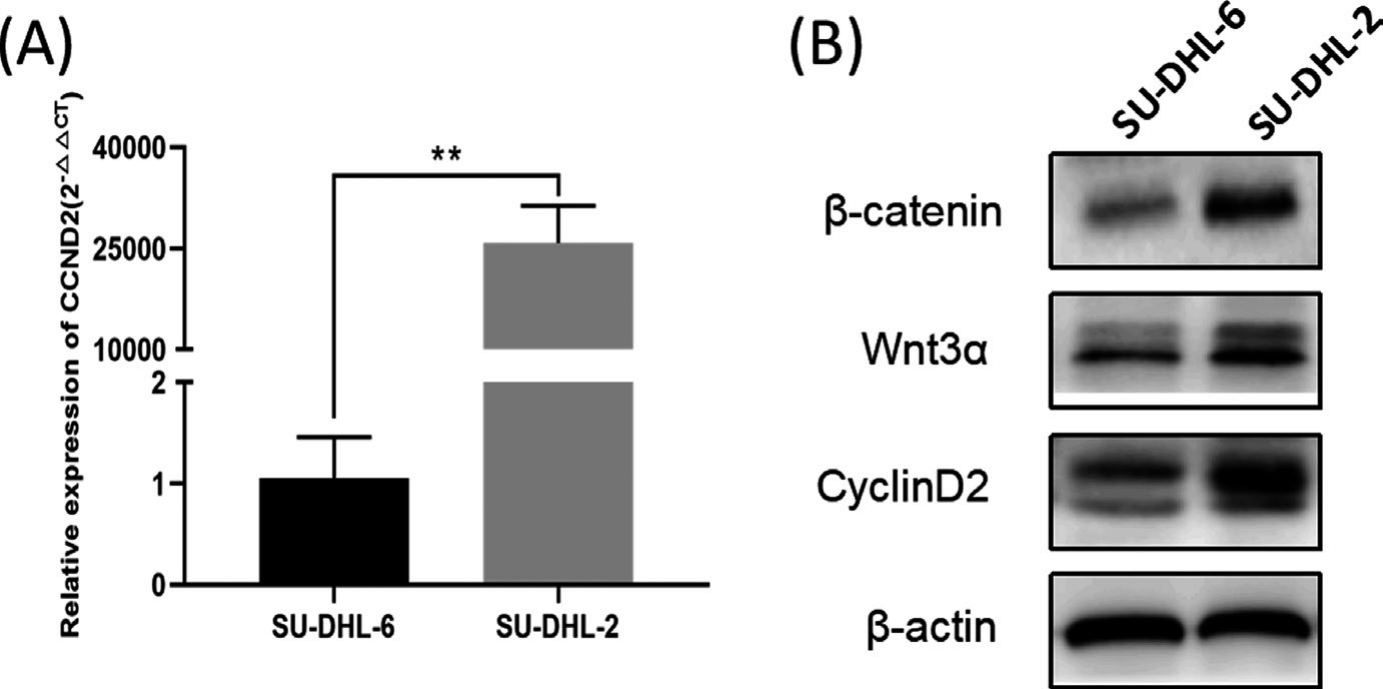
Total RNA and protein were extracted for qRT‐PCR and Western blot analysis. (A) The expression of CCND2 mRNA was measured by qRT‐PCR. (B) The expression of CCND2 and star proteins of WNT pathway were measured by Western blot. **p < 0.01

In this study, 2 cell lines of DLBCL (GCB subtype: SU‐DHL‐6, and ABC subtype: SU‐DHL‐2) were used to experimentally validate the biological information on the discovered hub genes. Our qRT‐PCR results indicated CCND2 was significantly upregulated in the ABC subtype (Figure 4A). To further explore the mechanism, we carried out the Western blot experiment. According to the enrichment results of the KEGG pathway, we selected the Wnt signal pathway. We found that the expression level of CCND2 positively correlated with the expression level of star protein molecules in the Wnt signaling pathway (Figure 4B).

## 4 DISCUSSION

**FIGURE 5:**
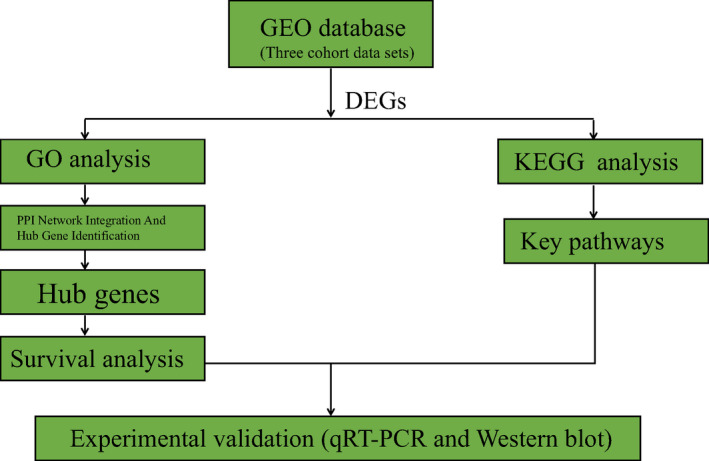
Workflow diagram

Diffuse large B‐cell lymphoma incidence is high and carries a significant disease burden. Within the DLBCL patients, two major DLBCL subtypes (GCB and ABC) have been defined by gene expression profiling.^16^ Patients with ABC‐DLBCL demonstrated worse outcomes than those with GCB‐DLBCL, with 5‐year OS rates ranging from 30% to 56% and 60% to 78%, respectively.^17^, ^18^ To our knowledge, this is the first report to analyze hub genes and key pathways using bioinformatics and basic experiments in two subtypes of DLBCL. The results showed their biological mechanisms were mainly associated with CCND2 and WNT pathway. These might provide a new strategy in the diagnosis and treatment of different subtypes of DLBCL (Figure 5).

Firstly, we used comprehensive bioinformatics analysis to explore potential vital genes and key pathways associated with ABC and GCB subtypes of DLBCL. Then, we performed some cell experiments to verify the most clinically significant gene and pathway. Finally, we found that ABC‐DLBCL had a significantly higher expression of star proteins in the Wnt/β‐catenin signaling pathway than GCB‐DLBCL. The increased expression level of CCND2 significantly affected the prognosis of patients. In this initial evaluation, we showed that overexpression of CCND2 and activation of the Wnt/β‐catenin signaling pathway were associated with poor survival in ABC‐DLBCL patients. Some literature has used bioinformatics methods to analyze the mechanisms of the two molecular subtypes of DLBCL, but there are some shortcomings compared with our research. For instance, Liu et al^6^ did not conduct experimental verification after bioinformatics prediction. In this study, three cohort datasets could provide us sufficient sample sizes (195 GCB and 169 ABC samples) to obtain potential candidate hub genes and key pathways more reliably. Not only that, we accessed many online publicly available databases like GO, KEGG, string database, and Cytoscape software to perform various analyzes. 10 hub genes were selected after using five calculation methods of the cytoHubba's application. Each algorithm included 10 unique hub genes. As a result, we obtained 3 common hub genes (IL‐10, CD44, CCND2) and performed a survival curve analysis on them. Furthermore, we found that high expression of CCND2 had a statistically significant effect on the prognosis of DLBCL patients, including OS and PFS. Our results were partly consistent with previous researches.^6^, ^19^ However, due to the difference in the source of the datasets, results from data on the same platform could still vary.^20^ Interestingly, the DEGs were mainly associated with "cell cycle" and "Wnt signaling pathway" in KEGG analysis. To verify the above results, our in‐depth experimental study focused on using two subtypes of DLBCL cell lines to detect the difference in RNA and protein expression levels in CCND2 and Wnt pathway. As a member of the Cyclin family, CCND2 (Cyclin D2) could activate cyclin‐dependent kinase 4(cdk4)/cdk6 to promote cell cycle progression.^21^ The abnormal expression of CCND2 may cause dysregulated cell proliferation. Even some studies showed that CCND2 methylation could promote tumor progression.^22^, ^23^, ^24^, ^25^ Wang et al^26^ concluded that high CCND2 mRNA expression negatively correlated with prognosis in patients with ABC‐DLBCL who received R‐CHOP. The result was consistent with ours. Although only CCND2 was statistically significant in the survival analysis for the three hub genes, it did not mean that the other genes were meaningless. For example, some scholars found that CSF (cerebrospinal fluid) IL‐10 levels could be a sensitive biomarker for differential diagnosis, detection of early recurrence, prognostic evaluation, and therapeutic strategy establishment in PCNSL (primary central nervous system lymphoma) cases.^27^ What's more, it had been reported that IL‐10 could promote tumor progression by promoting NF‐κB‐mediated transcription.^28^ CD44 is a transmembrane glycoprotein involved in B‐cell migration and activation, and it associates with the extracellular matrix changes that influence cell growth, survival, and differentiation.^29^ Higher CD44 expression has been noticed in ABC‐DLBCL than GCB‐DLBCL, but contradictory data have also been reported.^30^ Our data supported the results of Jelicic et al,^31^ who showed high CD44 expression in tumor cells correlates significantly with poorer EFS and OS. We also found that IL‐10 and CD44 were highly expressed in ABC‐DLBCL and conferred a poor prognosis. Although it had no statistically significant effect on the prognosis between the two subtypes of DLBCL, it may be related to the database sources, disease types, etc. Interestingly, we unexpectedly found that the Wnt pathway was better activated in ABC‐DLBCL. As is well‐known, the Wnt/β‐catenin pathway plays a prominent role in stem cell maintenance, embryonic development, and tumorigenesis.^32^ The cytoplasmic β‐catenin level determines the main function of Wnt/β‐catenin signaling. β‐catenin can mediate intracellular adhesion, differentiation, embryonic development, and tumorigenesis.^33^, ^34^ Our cell‐level experiments found that the RNA and protein expression of CCND2 in the ABC‐type cell line was significantly higher than that in the GCB‐type cell line and were directly proportional to the expressions of key proteins in the Wnt/β‐catenin pathway. Therefore, our study perhaps described and confirmed the hub genes and a key pathway of DLBCL subtypes to improve our understanding of the potential causes of differences in the clinical prognosis of DLBCL. Moreover, it remained exciting to the assessment of the prognosis of patients with changes in these genes.

However, this study also had shortcomings. One limitation was that we only used a single data source (GEO), other source like TCGA (The Cancer Genome Atlas) may also be need to confirm our results. In addition, our research focused just on the alterations of downstream protein levels of the disease mechanism. The upstream mechanism of CCND2 and Wnt pathway are also worthy of further exploration, such as different genetic and epigenetic changes. Fortunately, our findings may provide a direction for future in‐depth research.

Above all, we identified 120 DEGs between DLBCL GCB and ABC subtypes. Three common hub genes were found after applying five calculation methods of the CytoHubba's application, including IL‐10, CD44, and CCND2. The high expression of CCND2 had a statistically significant effect on the prognosis of DLBCL patients. The KEGG pathway analysis associated most of the DEGs with "cell cycle" and "Wnt signaling pathway". Experiments showed that the RNA and protein expression levels in CCND2 and Wnt pathway were different in ABC and GCB cell lines. These results might provide promising therapeutic targets or novel prognostic biomarkers of ABC and GCB subtypes of DLBCL.

## CONFLICT OF INTEREST

The authors declare that there is no conflict of interest regarding the publication of this paper.

## AUTHOR CONTRIBUTIONS

Q.L searched database informations, performed the experiments and wrote the manuscript. Y.M organized the figures and analyzed the statistics. L‐h.H interpreted the data. A.C and W‐w.Z collected relevant informations. Z‐m.Z designed and supervised the study, All authors are in agreement with the content of the manuscript for publication.

## Data Availability

The data generated or analyzed during this study are available from the corresponding author upon reasonable request.

## References

[1] Oiwa K , Fujita K , Lee S , et al. Utility of the geriatric 8 for the prediction of therapy‐related toxicity in older adults with diffuse large B‐cell lymphoma. Oncologist. 2021;26(3):215‐223. 33320984 10.1002/onco.13641 PMC7930418

[2] Bojarczuk K , Wienand K , Ryan JA , et al. Targeted inhibition of PI3Kalpha/delta is synergistic with BCL‐2 blockade in genetically defined subtypes of DLBCL. Blood. 2019;133(1):70‐80. 30322870 10.1182/blood-2018-08-872465 PMC6318426

[3] Maes A , Maes K , Vlummens P , et al. Maternal embryonic leucine zipper kinase is a novel target for diffuse large B cell lymphoma and mantle cell lymphoma. Blood Cancer J. 2019;9(12):87. 31740676 10.1038/s41408-019-0249-x PMC6861269

[4] Reddy A , Zhang J , Davis NS , et al. Genetic and functional drivers of diffuse large B cell lymphoma. Cell. 2017;171(2):481‐494. 28985567 10.1016/j.cell.2017.09.027 PMC5659841

[5] Zelenetz AD , Salles G , Mason KD , et al. Venetoclax plus R‐ or G‐CHOP in non‐Hodgkin lymphoma: results from the CAVALLI phase 1b trial. Blood. 2019;133(18):1964‐1976. 30850381 10.1182/blood-2018-11-880526 PMC6497517

[6] Liu Z , Meng J , Li X , et al. Identification of hub genes and key pathways associated with two subtypes of diffuse large B‐cell lymphoma based on gene expression profiling via integrated bioinformatics. Biomed Res Int. 2018;2018:3574534. 29992138 10.1155/2018/3574534 PMC5994323

[7] Sun S , Weile J , Verby M , et al. A proactive genotype‐to‐patient‐phenotype map for cystathionine beta‐synthase. Genome Med. 2020;12(1):13. 32000841 10.1186/s13073-020-0711-1 PMC6993387

[8] Risueño A , Hagner PR , Towfic F , et al. Leveraging gene expression subgroups to classify DLBCL patients and select for clinical benefit from a novel agent. Blood. 2020;135(13):1008‐1018. 31977005 10.1182/blood.2019002414 PMC7099333

[9] Williams PM , Li R , Johnson NA , Wright G , Heath JD , Gascoyne RD . A novel method of amplification of FFPET‐derived RNA enables accurate disease classification with microarrays. J Mol Diagn. 2010;12(5):680‐686. 20688907 10.2353/jmoldx.2010.090164 PMC2928433

[10] Dubois S , Tesson B , Mareschal S , Refining diffuse large B‐cell lymphoma subgroups using integrated analysis of molecular profiles. EBioMedicine. 2019;48:58‐69. 31648986 10.1016/j.ebiom.2019.09.034 PMC6838437

[11] Dybkær K , Bøgsted M , Falgreen S , et al. Diffuse large B‐cell lymphoma classification system that associates normal B‐cell subset phenotypes with prognosis. J Clin Oncol. 2015;33(12):1379‐1388. 25800755 10.1200/JCO.2014.57.7080 PMC4397280

[12] Huang DW , Sherman BT , Lempicki RA . Systematic and integrative analysis of large gene lists using DAVID bioinformatics resources. Nat Protoc. 2009;4(1):44‐57. 19131956 10.1038/nprot.2008.211

[13] Yu G , Wang LG , Han Y , He QY . clusterProfiler: an R package for comparing biological themes among gene clusters. OMICS. 2012;16(5):284‐287. 22455463 10.1089/omi.2011.0118 PMC3339379

[14] Franceschini A , Szklarczyk D , Frankild S , et al. STRING v9.1: protein‐protein interaction networks, with increased coverage and integration. Nucleic Acids Res. 2013;41:D808‐D815. 23203871 10.1093/nar/gks1094 PMC3531103

[15] Shannon P , Markiel A , Ozier O , et al. Cytoscape: a software environment for integrated models of biomolecular interaction networks. Genome Res. 2003;13(11):2498‐2504. 14597658 10.1101/gr.1239303 PMC403769

[16] Sha C , Barrans S , Cucco F , et al. Molecular high‐grade B‐cell lymphoma: defining a poor‐risk group that requires different approaches to therapy. J Clin Oncol. 2019;37(3):202‐212. 30523719 10.1200/JCO.18.01314 PMC6338391

[17] Klanova M , Sehn LH , Bence‐Bruckler I , et al. Integration of cell of origin into the clinical CNS International Prognostic Index improves CNS relapse prediction in DLBCL. Blood. 2019;133(9):919‐926. 30617197 10.1182/blood-2018-07-862862 PMC6396175

[18] Carpio C , Bouabdallah R , Ysebaert L , et al. Avadomide monotherapy in relapsed/refractory DLBCL: safety, efficacy, and a predictive gene classifier. Blood. 2020;135(13):996‐1007. 31977002 10.1182/blood.2019002395 PMC7099331

[19] Luo B , Gu YY , Wang XD , Chen G , Peng ZG . Identification of potential drugs for diffuse large b‐cell lymphoma based on bioinformatics and Connectivity Map database. Pathol Res Pract. 2018;214(11):1854‐1867. 30244948 10.1016/j.prp.2018.09.013

[20] Loeffler‐Wirth H , Kreuz M , Hopp L , et al. A modular transcriptome map of mature B cell lymphomas. Genome Med. 2019;11(1):27. 31039827 10.1186/s13073-019-0637-7 PMC6492344

[21] Dick FA , Goodrich DW , Sage J , Dyson NJ . Non‐canonical functions of the RB protein in cancer. Nat Rev Cancer. 2018;18(7):442‐451. 29692417 10.1038/s41568-018-0008-5 PMC6693677

[22] Li WC , Wu YQ , Gao B , Wang CY , Zhang JJ . MiRNA‐574‐3p inhibits cell progression by directly targeting CCND2 in colorectal cancer. Biosci Rep. 2019;39(12):BSR20190976. 31729531 10.1042/BSR20190976 PMC6911158

[23] He X , Chen SY , Yang Z , et al. miR‐4317 suppresses non‐small cell lung cancer (NSCLC) by targeting fibroblast growth factor 9 (FGF9) and cyclin D2 (CCND2). J Exp Clin Cancer Res. 2018;37(1):230. 30227870 10.1186/s13046-018-0882-4 PMC6145328

[24] Qian Y , Wang JW , Fang Y , et al. Measurement of Cyclin D2 (CCND2) gene promoter methylation in plasma and peripheral blood mononuclear cells and alpha‐fetoprotein levels in patients with hepatitis B virus‐associated hepatocellular carcinoma. Med Sci Monit. 2020;26:e927444. 33320844 10.12659/MSM.927444 PMC7749526

[25] Park SY , Lee CJ , Choi JH , et al. The JAK2/STAT3/CCND2 Axis promotes colorectal cancer stem cell persistence and radioresistance. J Exp Clin Cancer Res. 2019;38(1):399. 31511084 10.1186/s13046-019-1405-7 PMC6737692

[26] Wang D , Zhang Y , Che YQ . CCND2 mRNA expression is correlated with R‐CHOP treatment efficacy and prognosis in patients with ABC‐DLBCL. Front Oncol. 2020;10:1180. 32850340 10.3389/fonc.2020.01180 PMC7396626

[27] Geng M , Song Y , Xiao H , et al. Clinical significance of interleukin‐10 concentration in the cerebrospinal fluid of patients with primary central nervous system lymphoma. Oncol Lett. 2021;21(1):2. 33240408 10.3892/ol.2020.12263 PMC7681207

[28] Carr M , Mamand S , Chapman KL , Perrior T , Wagner SD . IKKepsilon and TBK1 in diffuse large B‐cell lymphoma: a possible mechanism of action of an IKKepsilon/TBK1 inhibitor to repress NF‐kappaB and IL‐10 signalling. J Cell Mol Med. 2020;24(19):11573‐11582. 32858764 10.1111/jcmm.15774 PMC7576278

[29] Harley JB , Chen X , Pujato M , et al. Transcription factors operate across disease loci, with EBNA2 implicated in autoimmunity. Nat Genet. 2018;50(5):699‐707. 29662164 10.1038/s41588-018-0102-3 PMC6022759

[30] Wei X , Xu M , Wei Y , et al. The addition of rituximab to CHOP therapy alters the prognostic significance of CD44 expression. J Hematol Oncol. 2014;7:34. 24739401 10.1186/1756-8722-7-34 PMC4022142

[31] Jelicic J , Balint MT , Jovanovic MP , et al. The role of lymphocyte to monocyte ratio, microvessel density and HiGH CD44 tumor cell expression in non Hodgkin lymphomas. Pathol Oncol Res. 2016;22(3):567‐577. 26750138 10.1007/s12253-015-0032-7

[32] Tsukiyama T , Zou J , Kim J , et al. A phospho‐switch controls RNF43‐mediated degradation of Wnt receptors to suppress tumorigenesis. Nat Commun. 2020;11(1):4586. 32934222 10.1038/s41467-020-18257-3 PMC7492264

[33] Kim M , Kim S , Lee SH , et al. Merlin inhibits Wnt/beta‐catenin signaling by blocking LRP6 phosphorylation. Cell Death Differ. 2016;23(10):1638‐1647. 27285107 10.1038/cdd.2016.54 PMC5041192

[34] Jiang S , Kong P , Liu X , Yuan C , Peng K , Liang Y . LncRNA FLVCR1‐AS1 accelerates osteosarcoma cells to proliferate, migrate and invade via activating wnt/beta‐catenin pathway. J BUON. 2020;25(4):2078‐2085. 33099956

